# Postoperative pain after different doses of remifentanil infusion during anaesthesia: a meta-analysis

**DOI:** 10.1186/s12871-023-02388-3

**Published:** 2024-01-13

**Authors:** Xinyi Huang, Jinxia Cai, Zhu Lv, Zijun Zhou, Xiaotian Zhou, Qimin Zhao, Jiehao Sun, Long Chen

**Affiliations:** 1 Centre for Rehabilitation Medicine, Department of Anaesthesiology, Zhejiang Provincial People’s Hospital (Affiliated People’s Hospital, Hangzhou Medical College), Hangzhou, Zhejiang China; 2 Department of Anaesthesiology, 1st affiliated hospital, Wenzhou Medical University, Ouhai District, Wenzhou, Zhejiang China

**Keywords:** Remifentanil, Dose-dependent, Hyperalgesia, Allodynia, Postoperative

## Abstract

**Background:**

This meta-analysis aimed to explore the correlation between the different doses of remifentanil-based anaesthesia and postoperative pain in randomised trials.

**Methods:**

The electronic databases including PubMed, Cochrane, clinical trial registries, and Google Scholar were searched up to November 2022 for randomised controlled trials (RCTs) that assessed the dose dependent efficacy of remifentanil for postoperative pain intensity and hyperalgesia.

**Results:**

31 studies involving 2019 patients were included for analysis. Compared with the high remifentanil dose administration, patients in low doses showed less postoperative pain intensity at 1-2 h (weighted mean differences (WMD): 0.60, 95% CI, 0.05 to 1.15), 3-8 h (WMD: 0.38, 95% CI, 0.00 to 0.75), 24 h (WMD: 0.26, 95% CI, 0.04 to 0.48) and 48 h (WMD: 0.32, 95% CI, 0.09 to 0.55). Remifentanil-free regimen failed to decrease the pain score at 24 h (WMD: 0.10, 95% CI, -0.10 to 0.30) and 48 h (WMD: 0.15, 95% CI, -0.22 to 0.52) in comparison with remifentanil-based anaesthesia. After excluding trials with high heterogeneity, the dose of the remifentanil regimen was closely correlated with the postoperative pain score (*P*=0.03). In addition, the dose of the remifentanil regimen was not associated with the incidence of postoperative nausea and vomiting (PONV) (*P*=0.37).

**Conclusions:**

Our meta-analysis reveals that the low dose of remifentanil infusion is recommendable for general anaesthesia maintenance. No evidence suggests that remifentanil-free regimen has superiority in reducing postoperative pain. Moreover, remifentanil doesn’t have a dose dependent effect in initiating PONV.

**Trial registration:**

The protocol of present study was registered with PROSPERO (CRD42022378360).

**Supplementary Information:**

The online version contains supplementary material available at 10.1186/s12871-023-02388-3.

## Introduction

Opioids are commonly used to alleviate perioperative pain during surgery. However, opioid, especially remifentanil use, can cause opioid tolerance and induce paradoxical pain [1]. Remifentanil was associated with primary and secondary hyperalgesia and can lead to opioid addiction.

In recent years, opioid-free general anaesthesia has been introduced to avoid unexpected pain. Opioid-free anaesthesia using dexmedetomidine or propofol [2–4] has been associated with less postoperative pain, resulting in less postoperative opioid consumption. However, the absence of remifentanil or other opioids during the surgery increases the amount of sedative infusion and results in delayed recovery [5].

It was found that remifentanil had a dose-dependent correlation with postoperative pain threshold [6, 7]. However, these findings are contrary to those of other studies that did not show an effect of the intraoperative opioid dose on postoperative pain intensity and rescue morphine consumption [8]. There is still a pending question about whether remifentanil infusion should be abandoned. There are a limited number of studies that have evaluated different doses of remifentanil and their relationship to postoperative pain intensity.

This meta-analysis aimed to evaluate dose dependent effect of remifentanil on the postoperative analgesic effect, secondary hyperalgesia, and side effects after general anaesthesia.

## Materials and methods

This meta-analysis of randomised, controlled trials (RCTs) was performed in accordance with the guidelines of the Preferred Reporting Items for Systematic Reviews and Meta-Analyses (PRISMA) statement (Additional file 1). The protocol was registered with PROSPERO (CRD42022378360).

### Eligibility criteria

Inclusion Criteria: randomised controlled trials were based on remifentanil anaesthesia or remifentanil free anaesthesia and focused on postoperative pain intensity and hyperalgesia in adults.

In order to exclude the impact of dexmedetomidine, the trials with dexmedetomidine only used in the remifentanil-free group were not included in the meta-analysis. So the exclusion criteria were as follows: dexmedetomidine was only applied in remifentanil-free group, general anaesthesia with epidural analgesia or nerve block, observational studies, non-randomised controlled trials, studies published as abstracts, duplicate articles, populations with chronic opioid use, and articles reporting no indispensable data.

### Search strategy

PubMed, Cochrane, clinical trial registries, and Google Scholar were searched to retrieve studies published up to November 2022 without language restrictions (by XH and JS). The following search string was used ("remifentanil" OR "remifentanyl" OR "opioid" OR "opiate") AND ("hyperalgesia" OR "hyperalgesia" OR "hyperalgesias" OR "hyperanalgesia" OR "nociception" OR "nociceptive" OR "pronociception" OR "pronociceptive" OR "allodynia" OR "tolerance") (Full links are given in Additional file 2). The searches were limited to human trials. A manual search of the references listed in the reports and reviews was performed. We reviewed the trial registries when available. In the case of secondary publications, the original papers were reviewed.

### Selection of included studies

Three reviewers (XH, JC, and ZL) independently screened the titles and abstracts obtained by the literature search. The remaining full texts were independently retrieved and evaluated by the authors to determine whether the retrieved trials met the inclusion criteria. Disagreements were discussed among the investigators to reach a consensus.

### Data extraction

The following data were extracted from the included studies: participant demographics, type of surgery, anaesthetic selection, intraoperative remifentanil regimens, pain scores at all reported times, postoperative allodynia, time to the first analgesic request, and opioid-related side effects. Pain scores on different scales were converted to a standardized 0-10 analogue scale. Any differences resulting from discrepant assessments during data extraction and analysis were resolved through discussion among the study authors. Data reported in the form of a graph were extracted with the assistance of graphics processing software (Web plot Digitalise, HTML5 Software, University of Notre Dame, USA).

### Postoperative outcomes

Primary outcome: Pain score at 1-2, 3-8, 24, and 48 h postoperatively.

Secondary outcomes: Periincisional wound allodynia and forearm allodynia, time to first postoperative analgesic requirement, postoperative consumption of rescue analgesics in milligrams of morphine equivalence, postoperative nausea and vomiting (PONV), and postoperative shivering.

Assessment of Methodological Quality and Risk of Bias:

The risk of bias was independently assessed using the Cochrane Collaboration tool [9]. Studies with a dropout rate of less than 20% were considered “low-risk” of attrition bias; otherwise, they were assessed as “high risk of bias”. “Other potential sources of bias” were assessed as high-risk in studies that had fewer than 15 participants per arm. However, there is currently no consensus on the trial size in this setting.

### Data synthesis and analysis

Meta-analyses were performed with the assistance of Review Manager 5.4 (Nordic Cochrane Centre, The Cochrane Collaboration, Copenhagen, Denmark), Comprehensive Meta-analysis version 2.2.034 (Biostat, USA), Trial Sequential Analysis Viewer version 0.9.5.5 Beta (Copenhagen Trial Unit, 2016) and STATA 15.0 (STATA CORP, Texas, USA).

For trials that did not report the results in the form of mean ± standard deviation (SD), the corresponding authors were contacted thrice by mail to supply the missing data. If no response was obtained, the sample size (n), median (m), minimum value (a), first quartile (q1), third quartile (q3), maximum value (b), were converted to mean ± SD by the specific formula [10, 11]. Note that the data may not always be given in full. The three frequently encountered scenarios are: C1 = {a, m, b; n}, C2 = {a, q1, m, q3, b; n}, C3 = {q1, m, q3; n}. The skew data can be diagnosed and transformed automatically based on the formular link: https://www.math.hkbu.edu.hk/~tongt/papers/median2mean.html.

We estimated the weighted mean differences (WMD) or standardised mean differences (SMD) with 95%CI for continuous data and the odds ratio (OR) for categorical data among the groups, with an overall estimate of the pooled effect. Forest plots were used to present the results graphically. Statistical heterogeneity across trials was assessed using the I^2^ value. A value of I^2^>50% or P<0.1 was considered as high heterogeneity. A random-effects model was applied in the case of high heterogeneity; otherwise, a fixed-effects model was adopted. For the primary outcome (pain score at postoperative 1-2, 3-8, 24 and 48 h), a priori sensitivity analysis was performed by removing the studies with a high risk of bias.

Mixed meta-regression was used to explore any potential dose-related interaction between the intraoperative remifentanil dose and postoperative pain intensity / PONV. In volunteers, remifentanil infusion at a rate of 0.10 μg/kg/min was reported [12] to provoke hyperalgesia, while opioid infusion at a rate of 0.05 μg/kg/min failed to induce RIH after discontinuation. The infusion rate at 0.1 µg/kg/min was proved to achieve a stable plasma concentration ranging between 2.7 and 2.9 ng/ml [13]. In addition, remifentanil plasma concentrations of 1.6 and 3.2 ng/ml correspond to steady-state concentrations achieved when infusing remifentanil at a constant rate of about 0.065 and 0.13 µg/kg/min. The result partially proved the linear correlation between the plasma concentration and constant infusion rate [14]. According to the linear formula, the trial which used a dose with 0.05 µg/kg/min was equally with the remifentanil concentrations of 1.2 ng/ml. Therefore, studies with remifentanil infusion less than 0.05 μg/kg/min or 1.2 ng/ml were allocated to the control group when performing meta-regression analysis. Based on the outcome of the mixed meta-regression analysis, piecewise linear regression was performed to define a cutoff value of the remifentanil dose to induce postoperative pain intensity.

Trial sequential analysis. In order to estimate the number of patients needed to allow for reliable statistical inference, we performed a sample size calculation to ensure that a sufficient number of patients were included in the meta-analysis. The random effect model using DerSimonian-Laird method was selected for the Trial sequential analysis program to integrate effective sizes. The required information size and the adjusted significance threshold for the postoperative pain score were calculated, with an anticipated 20% reduction of mean difference in pain score (mean difference=0.4) and variance of 0.4 with a 5% risk of type 1 error (β=0.8), and model variance-based heterogeneity correction.

### Assessment of publication bias

The risk of potential publication bias was evaluated using the Egger’s regression test.

## Results

**Fig. 1:**
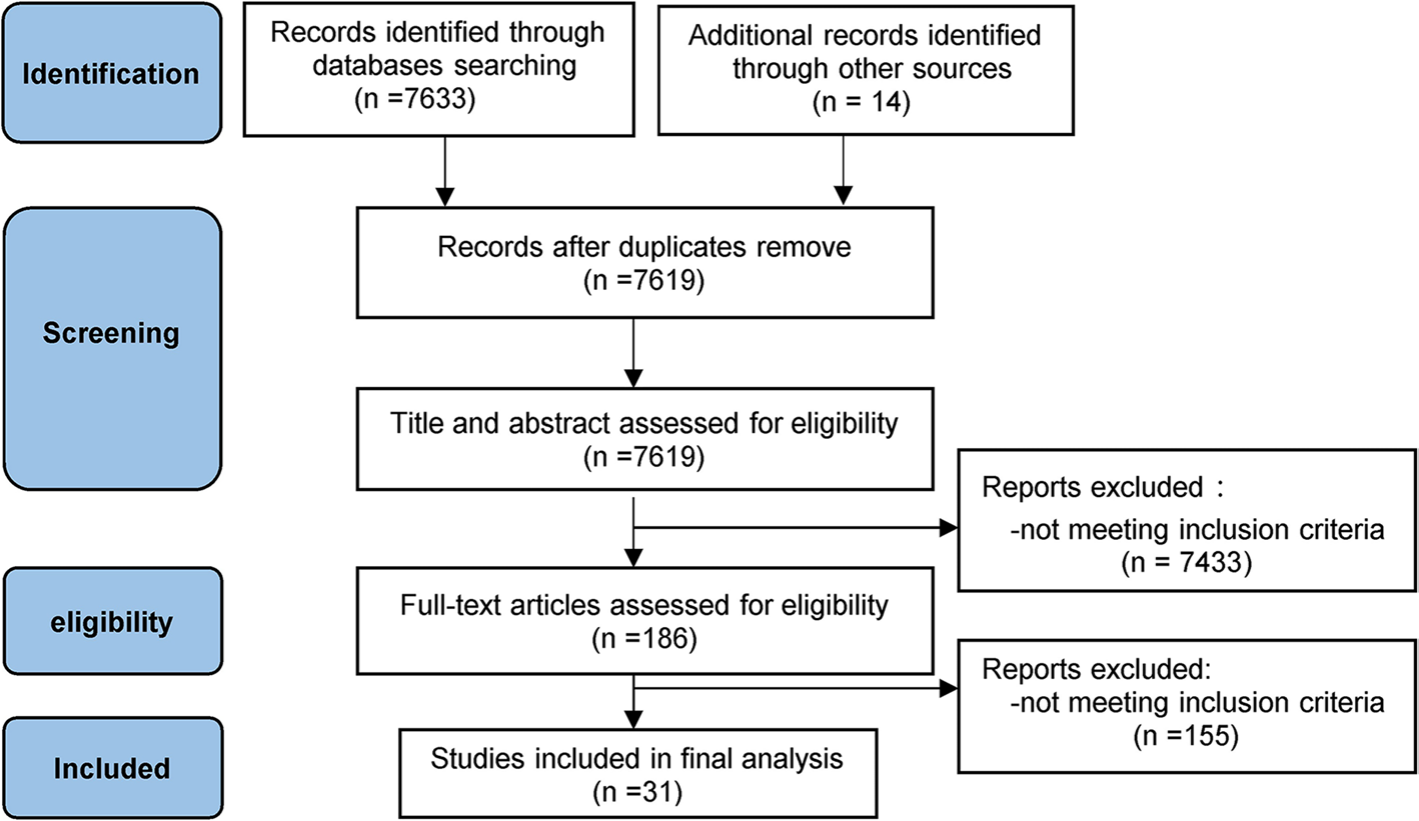
PRISMA Flow diagram

The literature search yielded 7633 results. 31 studies with a total of 2019 patients between 2000 and 2020 met our inclusion criteria, and were included in the meta-analysis [5–8, 15–41] (Fig. 1). No unpublished data were identified from clinical register or major annual meetings of anaesthesiology.

### Study characteristics

**Fig. 2:**
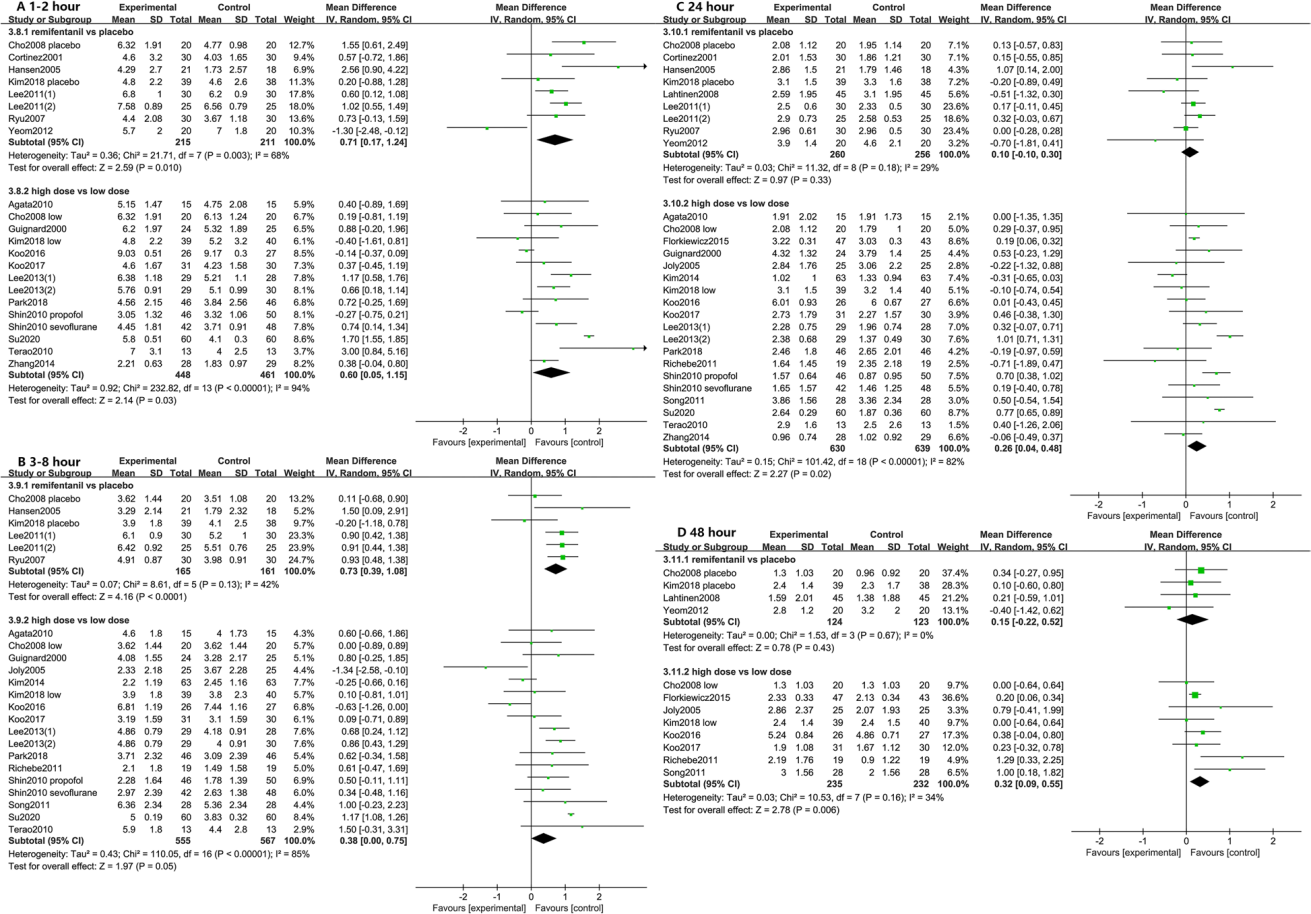
Forest plot for comparison of pain scores at 1-2 h (**A**), 3-8 h (**B**), 24 h (**C**), and 48 h (**D**). Data were pooled using a random-effects model to calculate the WMD and 95% CI for each outcome. CI indicates confidence interval; df, degrees of freedom; M-H, Mantel-Haenszel; WMD, weighted mean differences

4 studies were at a high risk of attrition bias [23, 32, 39, 40]. The other studies were all at low risk or unclear (Fig. 2). The characteristics of the included studies [5–8, 15–41] are shown in Table 1. The quality of the included studies is shown in Additional file 3, Additional file 4 and Table 2.

**Table 1:** Characteristics of included studies

Author	Surgery	Number	Remifentanil dose in the test groups	Anaesthesia maintenance	Postoperative analgesia	Outcome
Guignard2000 [15]	colorectal surgery	24/25	0.3 μg/kg/min vs 0.1 μg/kg/min	Desflurane	PCIA: morphine	①⑤⑦⑧⑨
Cortinez2001 [16]	gynaecological surgery	30/30	0.25 μg/kg/min vs placebo	Sevoflurane	PCIA: morphine	①⑦⑨
Sahin2004 [24]	lumbar discectomy	16/14	0.1 μg/kg/min vs placebo	Desflurane	PCIA: morphine	⑦
Joly2005 [17]	colorectal surgery	25/25	0.4 μg/kg/min vs 0.05 μg/kg/min	Desflurane	PCIA: morphine	①③⑤⑦⑧⑨
Hansen2005 [23]	abdominal surgery	21/18	0.4 μg/kg/min vs placebo	Sevoflurane	PCIA: morphine	①②⑦
Ryu2007 [30]	subtotal gastrectomy	30/30	1 ng/ml vs placebo	Sevoflurane	Not specified	①
Lahtinen2008 [18]	coronary bypass surgery	45/45	0.3 μg/kg/min vs placebo	Isoflurane + propofol	PCIA: oxycodone	①②⑤⑦⑧⑨
Cho2008 [5]	gynaecological surgery	20/20	3 ng/ml vs 1 ng/ml	Sevoflurane	PCIA: morphine	①⑦⑧
Cho2008 [5]	gynaecological surgery	20/20	3 ng/ml vs placebo	Sevoflurane	PCIA: morphine	①⑦⑧
Agata2010 [29]	orthognathic surgery	15/15	0.3 μg/kg/min vs 0.15 μg/kg/min	Sevoflurane	PCIA: fentanyl	①⑦⑨⑩
Terao2010 [21]	wrist arthrodesis	13/13	0.8 μg/kg/min vs 0.1 μg/kg/min	Sevoflurane	PCIA: fentanyl	①⑦
Shin2010 [19]	breast cancer surgery	88/98	4 ng/ml vs 1 ng/ml	Sevoflurane +propofol	PCIA: morphine	①⑦⑧⑨⑩
Lee2011(1) [27]	tonsillectomy	30/30	0.1 μg/kg/min vs placebo	Sevoflurane	i.v. pethidine/ketorolac	①⑤⑦
Lee2011(2) [26]	laparoscopic prostatectomy	25/25	0.3 μg/kg/min vs placebo	Desflurane	PCIA: morphine	①⑦⑨⑩
Richebe2011 [20]	coronary artery surgery	19/19	0.3 μg/kg/min vs 7 ng/ml	Propofol	PCIA i.v. morphine	①②③⑤⑦⑧⑨
Song2011[6]	thyroidectomy	28/28	0.2 μg/kg/min vs 0.05 μg/kg/min	Sevoflurane	i.v. fentanyl	①③④⑥⑧⑨⑩
Yeom2012 [25]	spinal fusion	20/20	0.03 μg/kg/min vs placebo	Sevoflurane	PCIA: fentanyl	①⑦⑨
Lee2013(1) [22]	laparoscopic hysterectomy	29/28	0.3 μg/kg/min vs 0.05 μg/kg/min	Desflurane	PCIA: morphine	①③⑤⑦⑧⑨⑩
Lee2013(2) [28]	laparoscopic urologic surgery	29/30	0.3 μg/kg/min vs 0.05 μg/kg/min	Desflurane	PCIA: morphine	①③⑤⑦⑨
Kim2014 [35]	local breast excision	63/63	10 ng/ml vs 5 ng/ml	Propofol	i.v. ketorolac	①⑨
Treskatsch2014 [39]	lower abdominal surgery	17/15	0.2 μg/kg/min vs 0.1 μg/kg/min	Sevoflurane	PCIA: morphine	⑤⑦⑧⑨
Zhang2014 [41]	thyroidectomy	28/29	1.2 μg/kg/min vs 0.2 μg/kg/min	Propofol	i.v. morphine	①④⑥⑦⑧⑨
Florkiewicz2015 [32]	cardiac surgery	47/43	0.3 μg/kg/min vs 0.1 μg/kg/min	Sevoflurane +propofol	PCIA: oxycodone	①②⑤⑦⑧⑨
Polat2015 [37]	nasal surgery	30/30	0.05 μg/kg/min vs placebo	Desflurane	i.v. fentanyl	①⑧
Koo2016 [8]	pancreaticoduodenectomy	26/27	4 ng/ml vs 1 ng/ml	Sevoflurane	PCIA: morphine	①②⑦⑨
Yamashita2016 [40]	gynaecological surgery	14/12	0.25 μg/kg/min vs 0.1 μg/kg/min	Sevoflurane	PCIA. fentanyl	⑤⑦⑧⑨
Koo2017 [7]	thyroid surgery	31/30	4 ng/ml vs 1 ng/ml	Desflurane	i.v. ketorolac	①③④⑥⑧⑨⑩
Kim2018 [34]	gastrectomy	39/40	12 ng/ml vs 2 ng/ml	Sevoflurane	PCIA: fentanyl	①②⑦⑧⑨
Kim2018 [34]	gastrectomy	39/38	12 ng/ml vs placebo	Sevoflurane	PCIA: fentanyl	①②⑦⑧⑨
Park2018 [36]	lower extremity surgery	46/46	0.25 μg/kg/min vs 0.05 μg/kg/min	Sevoflurane	i.v. pethidine, et al	①⑥⑦⑨⑩
Chang2019 [31]	tracheostomy	49/48	0.1 μg/kg/min vs placebo	Propofol	Not specified	①⑨
Khidr2020 [33]	cardiac surgery	23/25	3 ng/ml vs 1 ng/ml	Sevoflurane +propofol	PCIA: morphine	⑦⑧
Su2020 [38]	laparoscopic cholecystectomy	60/60	0.3 μg/kg/min vs 0.1 μg/kg/min	Sevoflurane	PCIA: sufentanil	①⑤⑦⑧⑨⑩

①Pain score at rest ②Pain score at movement ③Periincisional mechanical pain threshold ④Forearm mechanical pain threshold ⑤Time to first postoperative analgesic requirement ⑥Need for rescue analgesic ⑦Analgesic consumption ⑧Emergence time ⑨PONV ⑩Shivering

**Table 2:** Summary of findings and quality of evidence (GRADE)

Variable	No of Participants (studies)	Effect Size (95% CI)	Heterogeneity (I^2^)	Quality of Comments the Evidence (GRADE)
Pain score at 1-2 h				
remifentanil vs placebo	426(8 studies)	WMD 0.71(0.17, 1.24)	68%	⊕⊕^2,3^
high dose vs low dose	909(13 studies)	WMD 0.60(0.05, 1.15)	94%	⊕⊕⊕^3^
Pain score at 3-8 h				
remifentanil vs placebo	326(6 studies)	WMD 0.73(0.39, 1.08)	42%	⊕⊕⊕^2^
high dose vs low dose	1122(16 studies)	WMD 0.38(0.00, 0.75)	85%	⊕⊕⊕^3^
Pain score at 24 h				
remifentanil vs placebo	516(9 studies)	WMD 0.10(-0.10, 0.30)	29%	⊕⊕⊕^2^
high dose vs low dose	1269(18 studies)	WMD 0.26(0.04, 0.48)	82%	⊕⊕⊕^3^
Pain score at 48 h				
remifentanil vs placebo	247(4 studies)	WMD 0.15(-0.22, 0.52)	0%	⊕⊕⊕^1^
high dose vs low dose	467(8 studies)	WMD 0.32(0.09, 0.55)	34%	⊕⊕⊕⊕
periincisional wound allodynia				
high dose vs low dose	441(7 studies)	SMD -1.14(-1.47, -0.80)	61%	⊕⊕⊕^3^
forearm allodynia				
high dose vs low dose	174(3 studies)	SMD -0.46(-0.82, -0.10)	28%	⊕⊕⊕^1^
Analgesic consumption at 0-8 h				
remifentanil vs placebo	306(6 studies)	SMD 0.42(0.09, 0.75)	51%	⊕⊕^2,3^
high dose vs low dose	675(9 studies)	SMD 1.28(0.41, 2.16)	96%	⊕⊕⊕^3^
Analgesic consumption at 12 h				
high dose vs low dose	557(6 studies)	SMD 2.50(1.17, 3.83)	98%	⊕⊕⊕^3^
Analgesic consumption at 24-48 h				
remifentanil vs placebo	446(8 studies)	SMD 0.08(-0.31, 0.47)	75%	⊕⊕^2,3^
high dose vs low dose	1041(16 studies)	SMD 0.94(0.40, 1.49)	94%	⊕⊕⊕^3^
Time to first postoperative analgesic requirement				
remifentanil vs placebo	200(3 studies)	WMD-25.27(-32.09, -18.46)	72%	⊕^1,2,3^
high dose vs low dose	521(9 studies)	WMD -7.53(-10.31, -4.75)	31%	⊕⊕⊕⊕
PONV				
remifentanil vs placebo	414(6 studies)	OR 1.46(0.92, 2.32)	0%	⊕⊕⊕⊕
high dose vs low dose	1231(17 studies)	OR 1.13(0.87, 1.45)	9%	⊕⊕⊕⊕
Shivering				
high dose vs low dose	572(6 studies)	OR 3.98(2.59, 6.13)	0%	⊕⊕⊕⊕

*CI* Confidence interval, *GRADE* Grading of Recommendations Assessment, Development and Evaluation, *OR* Odds ratio, *PONV* Postoperative nausea and vomiting, *SM,* Standardized mean differences, *WMD* Weighted mean differences

The level of evidence was assessed by the GRADE method. ⊕⊕⊕⊕(High quality)**:** Further research is very unlikely to change our confidence in the estimate of effect. ⊕⊕⊕(Moderate quality)**:** Further research is likely to have an important impact on our confidence in the estimate of effect and may change the estimate. ⊕⊕(Low quality)**:** Further research is very likely to have an important impact on our confidence in the estimate of effect and is likely to change the estimate. ⊕(Very low quality)**:** We are very uncertain about the estimate.^1^ Downgraded for imprecision: optimal information size not reached. ^2^ Downgraded for insufficient data quality. ^3^ Downgraded for inconsistency (I^2^> 50%).

Remifentanil intervention was used in a wide range of surgical procedures. The doses of remifentanil administration ranged from 0.03 to 1.2 μg/kg/min. 22 trials compared high dose group with low-dose groups [5–8, 15, 17, 19–22, 28, 29, 32–36, 38–41]. 12 trials compared remifentanil with remifentanil-free groups [5, 16, 18, 23–27, 30, 31, 34, 37].

### Postoperative pain intensity

#### High vs. low dose of remifentanil administration

**Fig. 3:**
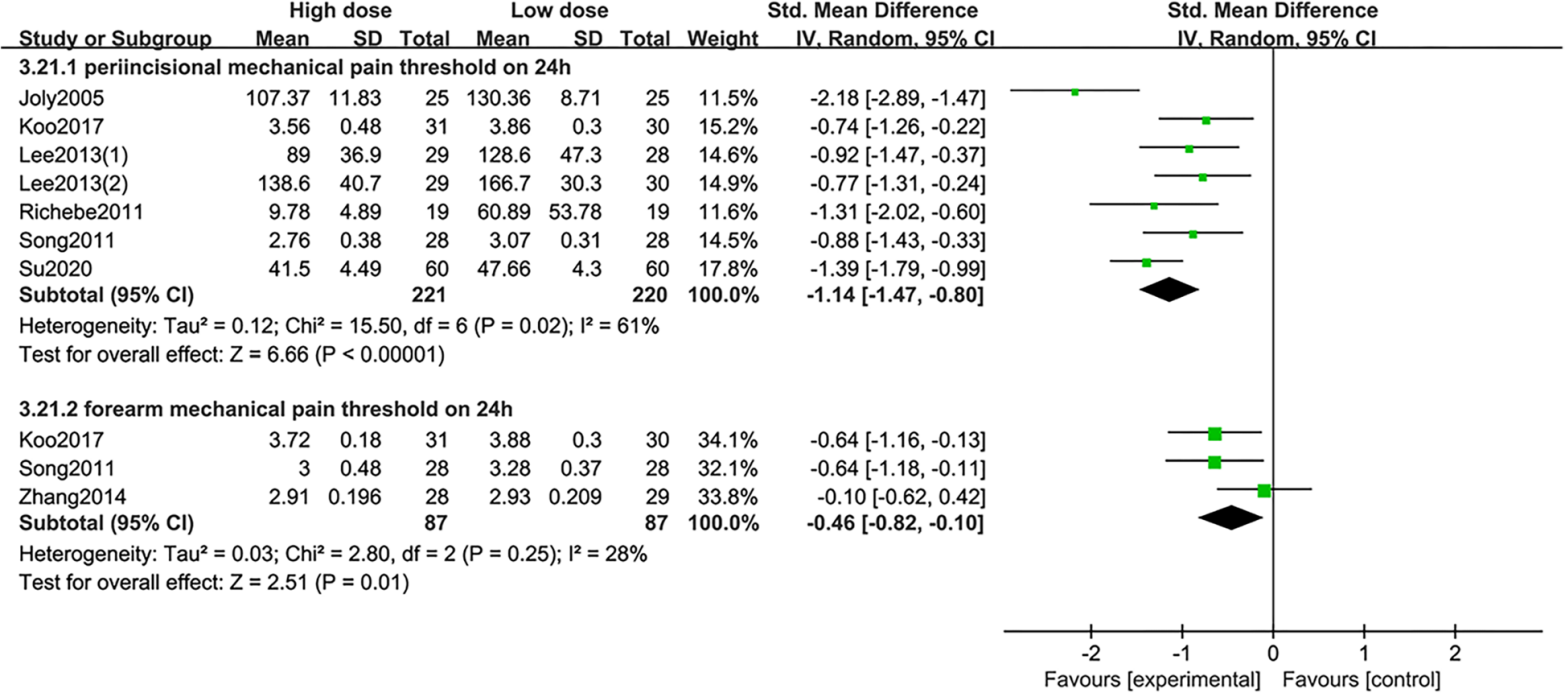
Forest plots for the periincisional wound and forearm allodynia. Data were pooled using a random-effects model to calculate the SMD and 95% CI for each outcome. Intervention refers to the high dose remifentanil, and control refers to the low dose remifentanil group. CI indicates confidence interval; df, degrees of freedom; M-H, Mantel-Haenszel; SMD, standardized mean differences

Compared with high-dose remifentanil administration, the postoperative pain score after low dose was significantly lower, including 1-2 h (909 participants in 13 studies, *P*=0.03) (Fig. 2A), 24 h (1269 participants in 18 studies, *P*=0.02) (Fig. 2C), and 48 h (467 participants in 8 studies, *P*=0.006) (Fig. 2D). There was no difference at 3-8 h (1122 participants in 16 studies, *P*=0.05) (Fig. 2B) between high- and low-dose group. Trial sequential analysis revealed that the pooled estimate on the primary endpoint exceeded the conventional and monitoring boundaries. We can conclude with sufficient statistical force that further studies will not modify the profile obtained with the meta-analysis on the primary endpoint. (Additional file 5). Low doses of remifentanil significantly inhibited postoperative pain and secondary hyperalgesia, characterised by a higher pain threshold for periincisional wound allodynia (441 participants in 7 studies, *P*<0.00001, I^2^: 61%, SMD: -1.14, 95% CI, -1.47 to -0.80) and forearm allodynia (174 participants in 3 studies, *P*=0.01, I^2^: 28%, SMD: -0.46, 95% CI, -0.82 to -0.10) (Fig. 3).

The time to the first postoperative analgesic requirement was prolonged in the low-dose remifentanil group compared to that in the high-dose group (521 participants in 9 studies, *P*<0.00001, WMD: -7.53, 95% CI, -10.31 to -4.75) (Table 3).

**Table 3:** Summary of findings by secondary outcomes

Variable	N	Effect Size (95% CI)	Heterogeneity (I^2^)
Analgesic consumption at 0-8 h			
remifentanil vs placebo	306	SMD 0.42(0.09, 0.75)	51%
high dose vs low dose	675	SMD 1.28(0.41,2.16)	96%
Analgesic consumption at 12 h			
high dose vs low dose	557	SMD 2.50(1.17,3.83)	98%
Analgesic consumption at 24-48 h			
remifentanil vs placebo	446	SMD 0.08(-0.31, 0.47)	75%
high dose vs low dose	1041	SMD 0.94 (0.40, 1.49)	94%
Time to first postoperative analgesic requirement			
remifentanil vs placebo	200	WMD -25.27(-32.09, -18.46)	72%
high dose vs low dose	521	WMD -7.53(-10.31, -4.75)	31%
PONV			
remifentanil vs placebo	414	OR 1.46(0.92, 2.32)	0%
high dose vs low dose	1231	OR 1.13(0.87, 1.45)	9%

*CI* Confidence interval, *OR* Odds ratio, *PONV* Postoperative nausea and vomiting, *SMD* Standardized mean differences, *WMD* Weighted mean differences

The postoperative analgesic requirement in the low-dose remifentanil group was less than that in the high-dose group at all time points, including the consumption at 0-8 h (675 participants in 9 studies, *P*=0.004, SMD: 1.28, 95% CI, 0.41 to 2.16), 0-12 h (557 participants in 6 studies, *P*=0.0002, SMD: 2.50, 95% CI, 1.17 to 3.83) and 24-48 h after surgery (1041 participants in 16 studies, *P*=0.0007, SMD: 0.94, 95% CI, 0.40 to 1.49) (Table 3).

#### Remifentanil-free vs. remifentanil regimen

The postoperative pain score in the remifentanil-free protocol showed superiority over the remifentanil regimen only at 1-2 h (426 participants in 8 studies, *P*=0.01) (Fig. 2A) and 3-8 h (326 participants in 6 studies, *P*<0.0001) (Fig. 2B). The study failed to detect the benefit of pain relief at 24 h (516 participants in 9 studies, *P*=0.33) (Fig. 2C) and 48 h (247 participants in 4 studies, *P*=0.43) (Fig. 2D). Correspondingly, postoperative analgesic requirements did not decrease in comparison with the remifentanil regimen during 24-48 h (446 participants in 8 studies, *P*=0.68, SMD: 0.08, 95% CI, -0.31 to 0.47) (Table 3).

The remifentanil-free protocol prolonged the time to the first postoperative analgesic requirement in comparison with the remifentanil regimen (200 participants in 3 studies, *P*<0.00001, WMD: -25.27, 95% CI, -32.09 to -18.46) (Table 3).

#### Bias of publication

The Egger linear regression test indicated no evidence of publication bias for postoperative pain intensity (at 1-2 h: *P*=0.078; 3-8 h: *P*=0.058; 24 h: *P*=0.633; 48 h: *P*=0.612) (Additional file 6).

Sensitivity analysis: After exclusion of four studies [23, 32, 39, 40] with a high risk of bias, the remaining 27 studies were robust to post hoc sensitivity analysis. The pain score in the low-dose remifentanil regimen still showed superiority over the high-dose group at all time points, including at 1-2 h (*P*=0.03, I^2^: 94%, WMD: 0.60, 95% CI, 0.05 to 1.15), 3-8 h (*P*=0.05, I^2^: 85%, WMD: 0.38, 95% CI, 0.00 to 0.75), 24 h (*P*=0.04, I^2^: 79%, WMD: 0.26, 95% CI, 0.01 to 0.51), and 48 h (*P*=0.01, I^2^: 34%, WMD: 0.40, 95% CI, 0.09 to 0.72). The remifentanil-free protocol still failed to detect the benefit of pain relief at 24 h (*P*=0.30, I^2^: 2%, WMD: 0.08, 95% CI, -0.07 to 0.24).

**Fig. 4:**
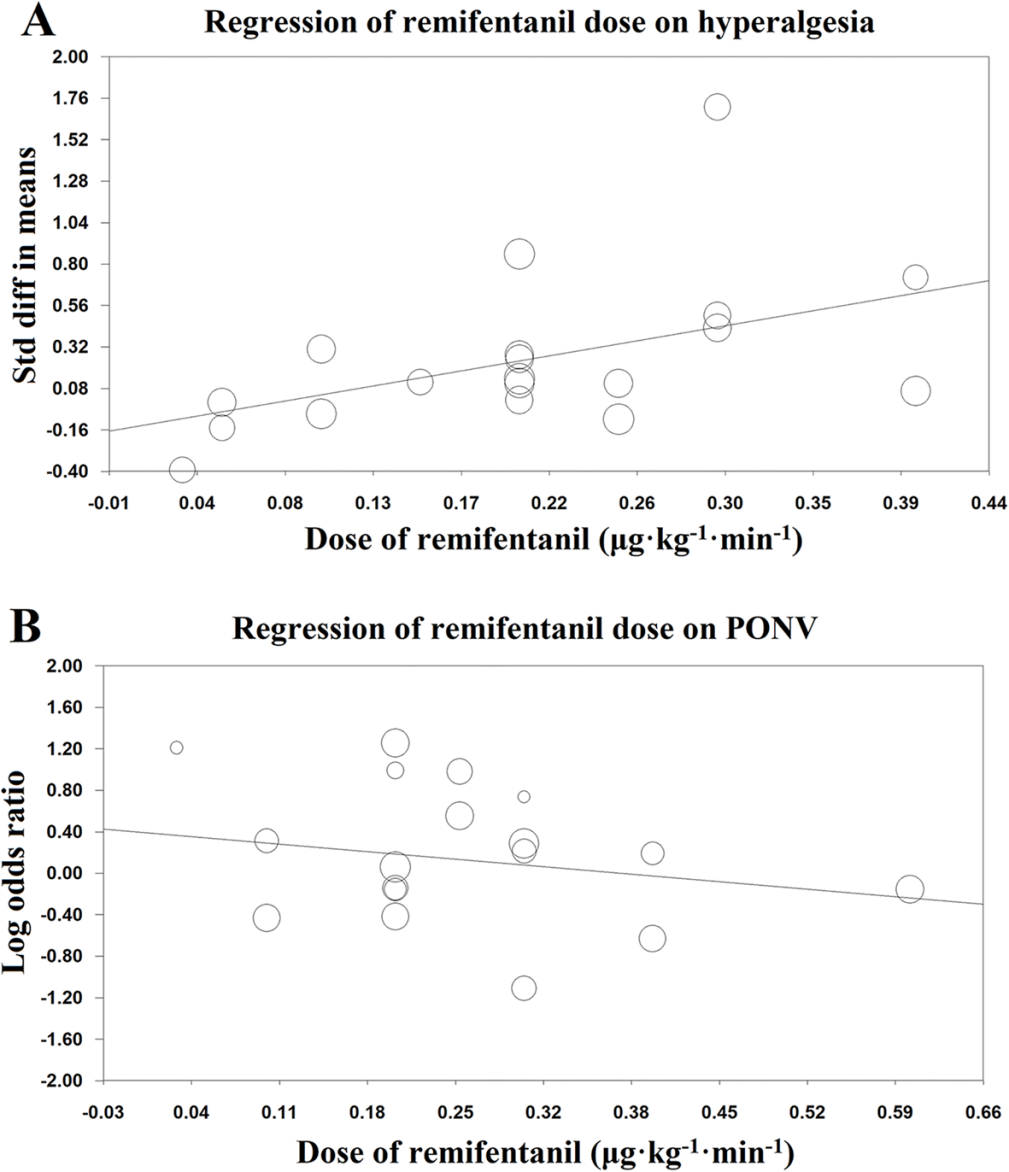
Mixed meta-regression to assess the interaction between remifentanil doses and hyperalgesia (**A**; *P*=0.03) and PONV (**B**; *P*=0.37) at 24 h. The size of the markers is proportional to the size of the study. PONV, postoperative nausea and vomiting; Std diff, standardized difference

After exclusion of studies with a high risk of bias, meta-regression analysis found that there was no association between remifentanil dose and the pain intensity at 24 h after the surgery (1264 participants in 17 studies, Tau^2^=0.12, slope of regression line: 0.39; *P*=0.57; 95% CI, -0.96 to 1.74) (Additional file 7). However, after excluding two trials [17, 18] with extreme data that generated high heterogeneity, the intensity of the pain score at 24 h was closely correlated with the dose of the intraoperative remifentanil infusion. (1085 participants in 15 studies, Tau^2^=0.09, slope of the regression line: 1.96; 95% CI, 0.19 to 3.72; *P*=0.03) (Fig. 4A). After piecewise linear regression analysis, the cutoff dose of the remifentanil to initiate postoperative pain was 0.1 μg/kg/min.

Subgroup analysis: The subgroup analysis is performed according to the surgery with high/low pain threshold: thoracotomy-laparotomy and non thoracotomy-laparotomy surgery. The pain score in the low-dose remifentanil regimen still showed superiority over the high-dose group at 24 h (thoracotomy-laparotomy: *P=*0.004, I^2^: 0%, WMD: 0.17, 95% CI, 0.05 to 0.28; non thoracotomy-laparotomy: *P=*0.02, I^2^: 82%, WMD: 0.35, 95% CI, 0.06 to 0.65). The remifentanil-free protocol still failed to detect the benefit of pain relief at 24 h (thoracotomy-laparotomy: *P=*0.75, I^2^: 30%, WMD: 0.05, 95% CI, -0.25 to 0.35; non thoracotomy-laparotomy: *P=*0.26, I^2^: 34%, WMD: 0.17, 95% CI, -0.13 to 0.47).

### Secondary outcomes

#### PONV

Neither remifentanil-free nor low-dose remifentanil exposure showed superiority in inhibiting the incidence of PONV (Table 3). There was no association between the dose of remifentanil infusion and the incidence of PONV (1142 participants in 14 studies, Tau^2^=0.09, slope of regression line: -1.06; 95% CI, -3.39 to 1.28; *P*=0.37) (Fig. 4B).

#### Shivering

Compared with the high-dose group, the low-dose group effectively suppressed the incidence of shivering (572 participants in 6 studies, *P*<0.00001, I^2^: 0%, OR: 3.98, 95% CI, 2.59 to 6.13).

## Discussion

In this study, low dose of remifentanil was correlated with lower pain score and less allodynia. Compared with the remifentanil regimen, the remifentanil-free group showed no benefit in inhibiting pain at 24 and 48 h. The meta-regression analysis found that the intensity of postoperative pain at 24 h was correlated with the dose of remifentanil infusion.

In a previous animal study [42], remifentanil ranged between 0.66 and 3.33 μg/kg/min has been reported to induce a dose-dependent pronociceptive effect. However, the drug concentrations were far exceeded the clinical demand. In the present study, the maximum remifentanil dose was 1.2 μg/kg/min. A previous meta-analysis [43, 44] with low certainty of evidence has shown that high doses of remifentanil are associated with acute pain after surgery. The use of opioid-free anaesthesia was reported to be associated with a reduction in PONV [45]. Moreover, neither of the papers [43–45] explored the dose-dependent association between remifentanil exposure and the incidence of postoperative pain or PONV. In the current meta-analysis that included more studies, quantitative and meta-regression analyses were introduced to conclude that the incidence of PONV was not correlated with the dose of remifentanil regimen, which was in contrast to a previous report [45].

A possible explanation for the higher pain score after remifentanil infusion is remifentanil-induced acute tolerance and hyperalgesia. The exact mechanism underlying opioid-induced hyperalgesia remains unclear. N-methyl-d-aspartate (NMDA) receptors have been shown to play a key role in opioid-induced hyperalgesia [46]. The reason for the inadequate postoperative pain control in the remifentanil protocol was attributed to NMDA activation. Sevoflurane, which is widely used in remifentanil-free groups, was reported to prevent central sensitisation through NMDA receptor antagonistic properties [46].

Opioid-induced thermal hyperalgesia can last for 2-7 days in rats [47]. Several studies have demonstrated that RIH occurred at 2 h and reached maximal at 24-48 h [47, 48]. In the present study, the higher pain scores in the high-dose remifentanil regimen lasted for 2 days after surgery.

Owing to the absence of opioid exposure, remifentanil-free anaesthesia can theoretically provide better resistance against the pathogenesis of hyperalgesia. Nevertheless, we found that there was no superiority over the remifentanil group in terms of postoperative pain scores at 24 and 48 h. Correspondingly, no improvement was detected in the rescue analgesic consumption at 24-48 h in the remifentanil-free group. Caution should be observed when using a remifentanil-free protocol in clinics.

The low-dose group inhibited postoperative pain during the first 48 h after surgery. Postoperative analgesic consumption, pain intensity and secondary hyperalgesia in the low-dose group were less evident than those in high-dose group. Notably, the degree of hyperalgesia was closely correlated with the amount of remifentanil infused. Compared with the remifentanil-free group, a small dose of remifentanil regimen seems recommendable.

The perioperative application of opioids is considered as a major factor in inducing PONV. The risk of PONV increases in direct proportion with the perioperative amount of opioid consumption [49]. Moreover, opioid-induced hyperalgesia requires more rescue opioids, which in turn aggravates PONV. A retrospective observational study [50] reported a dose-dependent association between the dose of intraoperative remifentanil and an increase in the risk of PONV. However, this meta-analysis upends our basic assumption. Regardless of remifentanil-free group or low-dose remifentanil regimen, the incidence of PONV did not decrease in comparison with the high-dose group. In other words, the incidence of PONV was not correlated with the amount of remifentanil infusion.

Postoperative shivering increases oxygen consumption, leading to an increased incidence of cardiovascular and neurological complications. It has been proposed that shivering results from rapid opioid withdrawal [51]. The present meta-analysis revealed an increased incidence of shivering after high dose of remifentanil.

### Limitation

Firstly, the decrease in pain scores (0-10) was on average less than one point if there was a comparison between remifentanil and remifentanil-free regimens or high and low doses of remifentanil regimens. Even if this was statistically significant, it is doubtful that the difference was clinically significant as expected. This will not restrain the use of higher intraoperative remifentanil regimen, especially with the proper use of intraoperative pain monitoring. Secondly, most of the included studies were conducted without using any nociception monitoring. In the absence of a monitoring device, such as the Nociception Level index, the remifentanil dose was not known to be adequate or insufficient during surgery. Furthermore, the low and high doses of opioids overlapped among the trials. Therefore, there is a high heterogeneity for most pain scores comparisons between high and low doses of remifentanil regimens. The meta-regression and sensitivity analysis were performed to exclude the impact of the high heterogeneity and correct selective bias. We believe that this limitation did not affect the validity of our results.

## Conclusion

Remifentanil-free anaesthesia has shown insufficient benefits in inhibiting postoperative pain. Patients receiving low dose remifentanil were correlated with lower pain scores, less allodynia and less shivering than those who received high dose remifentanil. In view of the current opioid epidemic, low-dose remifentanil anaesthesia should be recommended. These findings can be broadly generalised to patients across surgical disciplines.

### Supplementary Information

**Additional file 1.** PRISMA checklist.

**Additional file 2.** Search Strategy Based on initial PubMed Search.

**Additional file 3.** Risk of bias summary.

**Additional file 4.** Risk of bias assessment for included studies.

**Additional file 5.** Trial sequential analysis of pain score between the two different remifentanil doses at the 24 h. X-axis: the number of patients randomised; Y-axis: the cumulative Z-score; the blue cumulative Z-curve was constructed using a random-effects model. Red vertical line with diamonds: required information size of a meta-analysis.

**Additional file 6.** Publication bias for pain scores at 1-2 h (A; *P*=0.078), 3-8 h (B; *P*=0.058), 24 h (C; *P*=0.633), and 48 h (D; *P*=0.612) postoperatively.

**Additional file 7.** Mixed meta-regression (methods of moment) to assess the interaction between remifentanil dose equivalent and hyperalgesia at 24 h postoperatively before sensitivity analysis (*P*=0.57). The size of the markers is proportional to the size of the study. Std diff, standardized difference.

## Data Availability

Raw extracted data are available (on request) from the corresponding authors (LC and JS).

## Abbreviations

IQR: Interquartile range
OR: Odds ratio
NMDA: N-methyl-d-aspartate
PONV: Postoperative nausea and vomiting
PRISMA: Preferred Reporting Items for Systematic Reviews and Meta-Analyses
RIH: Remifentanil-induced hyperalgesia
SD: Standard deviation
SMD: Standardised mean differences
WMD: Weighted mean differences
95% CI: 95% confidence interval
