# Supplementary material for: Postoperative pain after different doses of remifentanil infusion during anaesthesia: a meta-analysis
Source: BMC Anesthesiol. 2024 Jan 13;24:25. doi: 10.1186/s12871-023-02388-3 (PMC10790271; doi:10.1186/s12871-023-02388-3)
Supplement: Supplementary file 2 — Additional file 2. Search Strategy Based on initial PubMed Search. [file 12871_2023_2388_MOESM2_ESM.docx]

Additional file 2. Search Strategy Based on initial PubMed Search

| Search Strategy |
| --- |
| ("remifentanil"[Title/Abstract] OR "remifentanyl"[Title/Abstract] OR "opioid"[Title/Abstract] OR "opiate"[Title/Abstract] OR "opioid-free"[Title/Abstract] OR "opiate-free"[Title/Abstract]) AND ("hyperalgesia"[MeSH Terms] OR "hyperalgesia"[All Fields] OR "hyperalgesias"[All Fields] OR "hyperanalgesia"[All Fields] OR ("nociception"[MeSH Terms] OR "nociception"[All Fields] OR "nociceptions"[All Fields] OR "nociceptive"[All Fields] OR "nociceptively"[All Fields]) OR ("pronociception"[All Fields] OR "pronociceptive"[All Fields]) OR ("hyperalgesia"[MeSH Terms] OR "hyperalgesia"[All Fields] OR "allodynia"[All Fields] OR "allodynias"[All Fields]) OR ("drug tolerance"[MeSH Terms] OR ("drug"[All Fields] AND "tolerance"[All Fields]) OR "drug tolerance"[All Fields]) OR ("withdraw"[All Fields] OR "withdrawal"[All Fields] OR "withdrawals"[All Fields] OR "withdrawing"[All Fields] OR "withdraws"[All Fields]))  https://pubmed.ncbi.nlm.nih.gov/?term=%28%22remifentanil%22%5BTitle%2FAbstract%5D+OR+%22remifentanyl%22%5BTitle%2FAbstract%5D+OR+%22opioid%22%5BTitle%2FAbstract%5D+OR+%22opiate%22%5BTitle%2FAbstract%5D+OR+%22opioid-free%22%5BTitle%2FAbstract%5D+OR+%22opiate-free%22%5BTitle%2FAbstract%5D%29+AND+%28%22hyperalgesia%22%5BMeSH+Terms%5D+OR+%22hyperalgesia%22%5BAll+Fields%5D+OR+%22hyperalgesias%22%5BAll+Fields%5D+OR+%22hyperanalgesia%22%5BAll+Fields%5D+OR+%28%22nociception%22%5BMeSH+Terms%5D+OR+%22nociception%22%5BAll+Fields%5D+OR+%22nociceptions%22%5BAll+Fields%5D+OR+%22nociceptive%22%5BAll+Fields%5D+OR+%22nociceptively%22%5BAll+Fields%5D%29+OR+%28%22pronociception%22%5BAll+Fields%5D+OR+%22pronociceptive%22%5BAll+Fields%5D%29+OR+%28%22hyperalgesia%22%5BMeSH+Terms%5D+OR+%22hyperalgesia%22%5BAll+Fields%5D+OR+%22allodynia%22%5BAll+Fields%5D+OR+%22allodynias%22%5BAll+Fields%5D%29+OR+%28%22drug+tolerance%22%5BMeSH+Terms%5D+OR+%28%22drug%22%5BAll+Fields%5D+AND+%22tolerance%22%5BAll+Fields%5D%29+OR+%22drug+tolerance%22%5BAll+Fields%5D%29+OR+%28%22withdraw%22%5BAll+Fields%5D+OR+%22withdrawal%22%5BAll+Fields%5D+OR+%22withdrawals%22%5BAll+Fields%5D+OR+%22withdrawing%22%5BAll+Fields%5D+OR+%22withdraws%22%5BAll+Fields%5D%29%29&filter=hum_ani.humans |
