# Supplementary figures and images for: Postoperative pain after different doses of remifentanil infusion during anaesthesia: a meta-analysis
Source: BMC Anesthesiol. 2024 Jan 13;24:25. doi: 10.1186/s12871-023-02388-3 (PMC10790271; doi:10.1186/s12871-023-02388-3)

Additional file 3. Risk of bias summary.


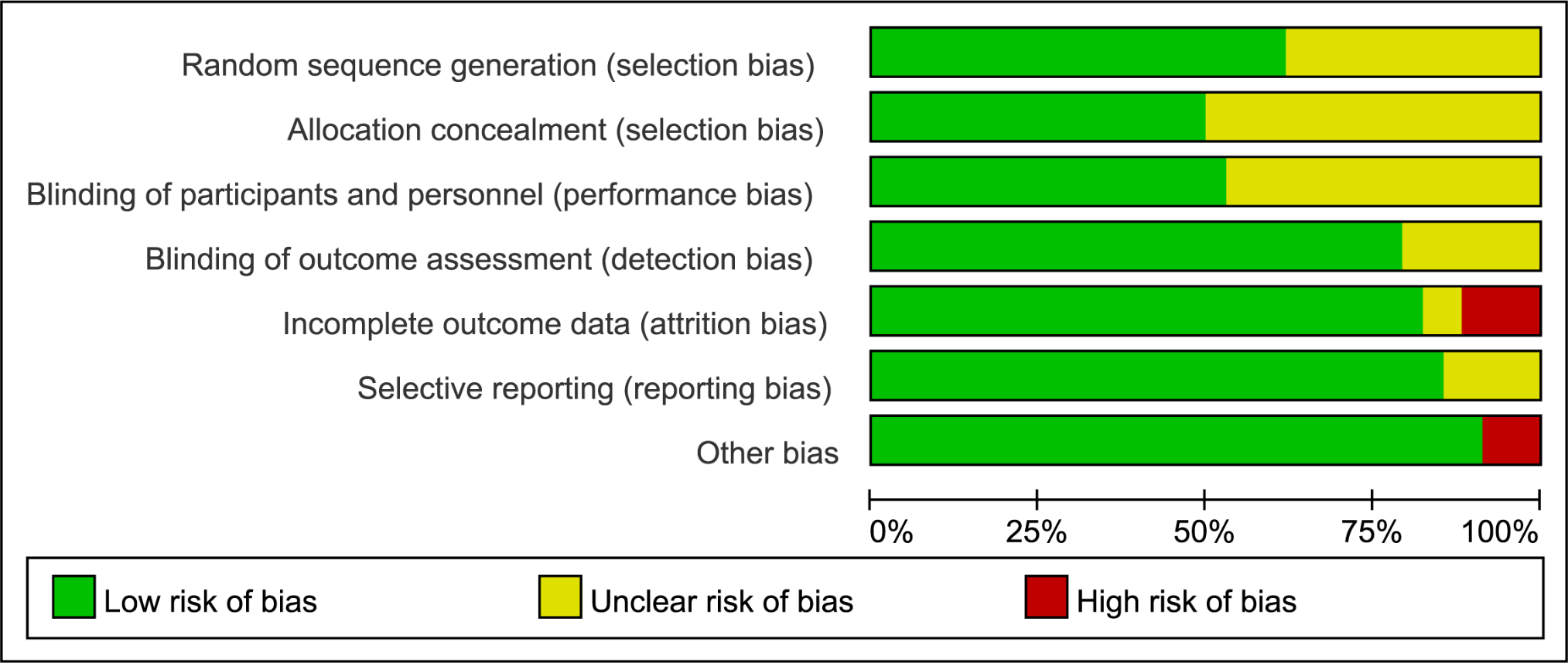

Supplement: Supplementary file 3 — Additional file 3. Risk of bias summary. [file 12871_2023_2388_MOESM3_ESM.docx]

Additional file 4. Risk of bias assessment for included studies.


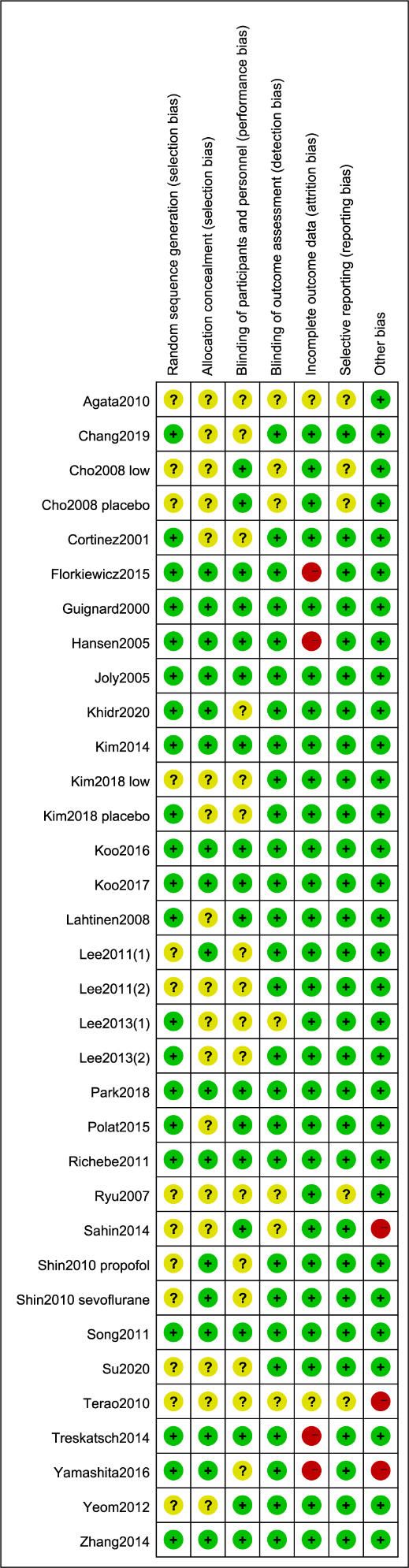

Supplement: Supplementary file 4 — Additional file 4. Risk of bias assessment for included studies. [file 12871_2023_2388_MOESM4_ESM.docx]

Additional file 6. Publication bias for pain scores at 1-2 h (A; *P*=0.078), 3-8 h (B; *P*=0.058), 24 h (C; *P*=0.633), and 48 h (D; *P*=0.612) postoperatively.


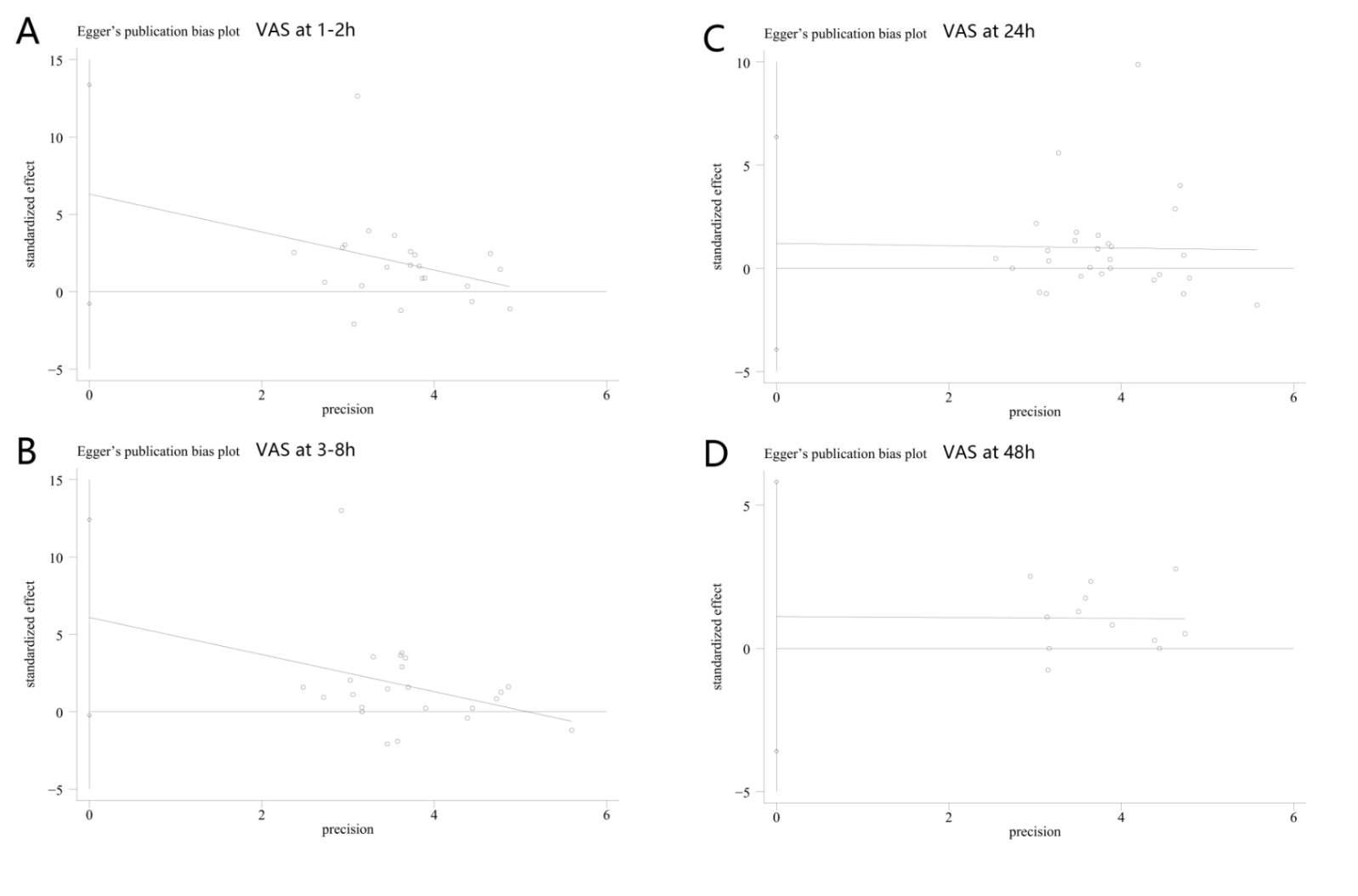

Supplement: Supplementary file 6 — Additional file 6. Publication bias for pain scores at 1-2 h (A; P=0.078), 3-8 h (B; P=0.058), 24 h (C; P=0.633), and 48 h (D; P=0.612) postoperatively. [file 12871_2023_2388_MOESM6_ESM.docx]
