# Supplementary material for: Postoperative pain after different doses of remifentanil infusion during anaesthesia: a meta-analysis
Source: BMC Anesthesiol. 2024 Jan 13;24:25. doi: 10.1186/s12871-023-02388-3 (PMC10790271; doi:10.1186/s12871-023-02388-3)

Additional file 5. Trial sequential analysis of pain score between the two different remifentanil doses at the 24 h.

X-axis: the number of patients randomised; Y-axis: the cumulative Z-score; the blue cumulative Z-curve was constructed using a random-effects model. Red vertical line with diamonds: required information size of a meta-analysis.


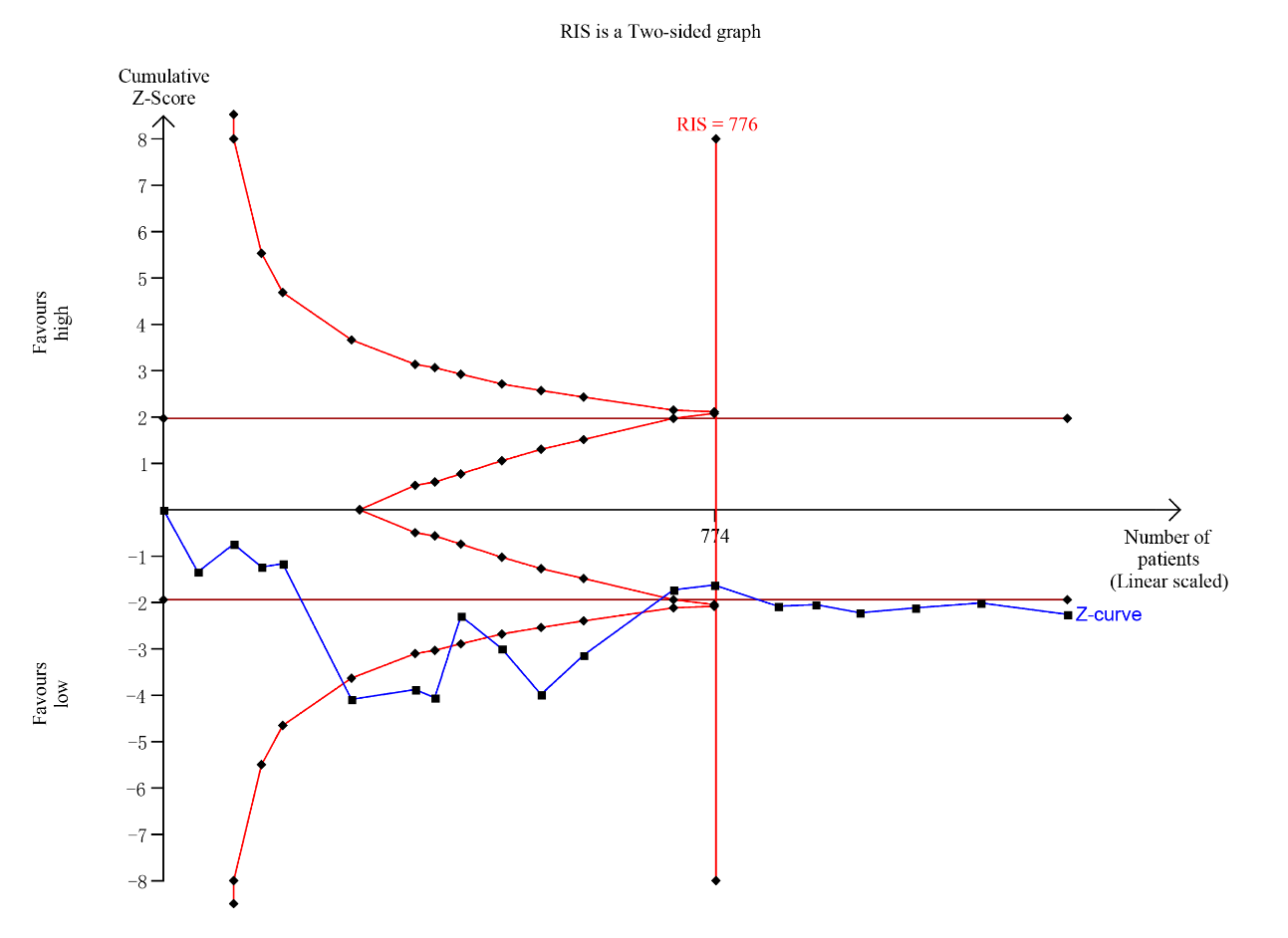

Supplement: Supplementary file 5 — Additional file 5. Trial sequential analysis of pain score between the two different remifentanil doses at the 24 h. X-axis: the number of patients randomised; Y-axis: the cumulative Z-score; the blue cumulative Z-curve was constructed using a random-effects model. Red vertical line with diamonds: required information size of a meta-analysis. [file 12871_2023_2388_MOESM5_ESM.docx]
