# Supplementary material for: Postoperative pain after different doses of remifentanil infusion during anaesthesia: a meta-analysis
Source: BMC Anesthesiol. 2024 Jan 13;24:25. doi: 10.1186/s12871-023-02388-3 (PMC10790271; doi:10.1186/s12871-023-02388-3)

Additional file 7. Mixed meta-regression (methods of moment) to assess the interaction between remifentanil dose equivalent and hyperalgesia at 24 h postoperatively before sensitivity analysis (*P*=0.57). The size of the markers is proportional to the size of the study. Std diff, standardized difference.


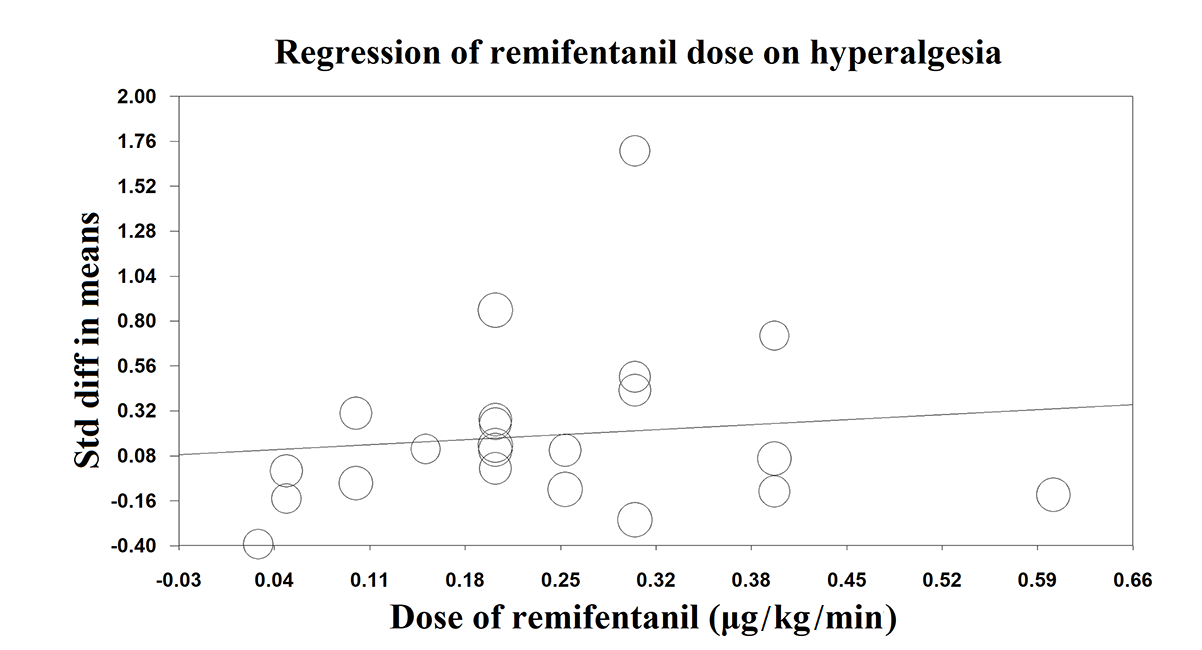

Supplement: Supplementary file 7 — Additional file 7. Mixed meta-regression (methods of moment) to assess the interaction between remifentanil dose equivalent and hyperalgesia at 24 h postoperatively before sensitivity analysis (P=0.57). The size of the markers is proportional to the size of the study. Std diff, standardized difference. [file 12871_2023_2388_MOESM7_ESM.docx]
